# Acute high temperature exposure impairs hypoxia tolerance in an intertidal fish

**DOI:** 10.1371/journal.pone.0231091

**Published:** 2020-04-02

**Authors:** Tristan J. McArley, Anthony J. R. Hickey, Neill A. Herbert

**Affiliations:** 1 Institute of Marine Science, University of Auckland, Leigh, New Zealand; 2 School of Biological Sciences, University of Auckland, Auckland, New Zealand; MARE - Marine and Environmental Sciences Centre, PORTUGAL

## Abstract

Acute heat shock has previously been shown to improve subsequent low O_2_ (hypoxia) tolerance in an intertidal fish species, a process known as cross-tolerance, but it is not known whether this is a widespread phenomenon. This study examined whether a rock pool specialist, the triplefin fish *Bellapiscis medius*, exhibits heat shock induced cross-tolerance to hypoxia, i.e., longer time to loss of equilibrium (LOE) and lower critical O_2_ saturation (S_*crit*_) after recovering from an acute heat challenge. Non-heat shock controls had a median time to loss of equilibrium (LOE_50_) of 54.4 min under severe hypoxia (7% of air saturation) and a S_*crit*_ of 15.8% air saturation. Contrary to expectations, however, treatments that received an 8 or 10°C heat shock showed a significantly shorter LOE_50_ in hypoxia (+8°C = 41.5 min; +10°C = 28.7 min) and no significant change in S_*crit*_ (+8°C = 17.0% air saturation; +10°C = 18.3% of air saturation). Thus, there was no evidence of heat shock induced cross-tolerance to hypoxia in *B*. *medius* because exposure to acute heat shock impaired hypoxia tolerance.

## Introduction

Marine organisms commonly have to contend with fluctuating environmental conditions (e.g. temperature, salinity, and oxygen) severe enough to elicit a physiological stress response. Moreover, environmental parameters rarely occur in isolation, so organisms must endure multiple environmental conditions that fluctuate either simultaneously or sequentially [1]. The response of organisms to multiple stressors can be complex and is largely dependent on the temporal pattern of exposure between each stressor. For example, when organisms experience multiple stressors simultaneously or in rapid succession, the combined negative effect is sometimes amplified (synergistic) relative to the additive effects of the individual stressors acting in isolation [1–3]. However, if there is a sufficient period of recovery between exposures, the combined negative effect of the stressors can be equal to the sum of the individual stressor effects (additive) or even reduced (antagonistic) [1,2]. In the case of an antagonistic response, exposure to one stressor can sometimes increase the tolerance of an organism to a second stressor; this phenomenon is known as cross-tolerance [4].

Due to the interaction between semi-diurnal tidal patterns and diel changes in air temperature and solar irradiance, certain physico-chemical parameters in intertidal rock pools tend to fluctuate out of phase and sequentially. Thus, for rock pool organisms such as intertidal fish, cross-tolerance could offer protection against different environmental stressors across consecutive tidal cycles. For example, temperature and oxygen availability in rock pools fluctuate widely, but when low tides roughly align with the middle of the day and night, peak daytime high temperatures occur out of phase with nocturnal hypoxia [1,5–9]. This is because the conditions associated with high temperature in isolated rock pools in the middle of the day (i.e. strong solar radiation) also promotes algal photosynthesis, which buffers against O_2_ depletion, and can even cause hyperoxia (O_2_ super-saturation). On the other hand, severe hypoxia can develop in isolated pools at night (i.e. only 8–11 h later) when respiring organisms draw down O_2_ at a time of comparatively stable temperatures [8]. Previous studies have demonstrated that high temperature exposure can induce cross-tolerance to hypoxia in fish. This includes examples where long-term (4–6 weeks) acclimation to a constant warm temperature results in an improved ability to tolerate hypoxia [10,11], and also examples where a one-off acute high temperature exposure (hours) has induced hypoxia cross-tolerance [12].

Rock pool habitats are prone to bouts of extreme high temperature followed by severe hypoxia, but only one study has addressed the tolerance of intertidal fish to this set of conditions [12]. Todgham et al., (2005) [12] showed that an intertidal species of sculpin (*Oligocottus maculosus*) had elevated tolerance (cross-tolerance) to both high salinity and hypoxia after recovering from an acute heat shock (2 h duration). With respect to measuring hypoxia cross-tolerance, these authors showed that fish exposed to an initial bout of acute heat shock had greater survival under subsequent exposure to severe hypoxia. It also appeared that cross-tolerance to high salinity could be maintained for quite a long period of time after heat shock (i.e. 8–48 h), and that the magnitude of heat shock was also important because high salinity cross-tolerance only developed with a +12°C heat shock and not a +15°C heat shock. Todgham et al. (2005) also examined the role of heat shock protein (Hsp) induction in conferring cross-tolerance. While these authors concluded that elevated Hsp were not required at the time of exposure to a second stressor to confer cross-tolerance, there was evidence that an initial heat shock primed the gills to respond to high salinity and the liver to hypoxia with an altered Hsp response. As 8 h was the minimum recovery time for cross-tolerance to develop in *O*. *maculosus*, and this period matched the approximate time between successive periods of low tide emersion in the intertidal zone, it was suggested that a protective effect of cross-tolerance might be relevant for fish under real life conditions [12].

In order to build upon the findings of Todgham et al., (2005) [12], the present study examined cross-tolerance in another specialist intertidal fish (*Bellapiscis medius*, Günther, 1861) to assess whether heat shock induced cross-tolerance is a common response in rock pool fishes. To assess the presence of cross-tolerance in *B*. *medius*, the hypoxia tolerance of this species was assessed after a ~19 h period of recovery from either a +8°C (21–29°C) or +10°C (21–31°C) heat shock treatment over a 5 h ramping period. Todgham et al. (2005) [12] observed a peak in cross-tolerance 12–24 h following exposure to heat shock in *O*. *maculosus*; thus, as cross-tolerance was only assessed at one time point in the current study, we chose a recovery time (19 h) within the timeframe of peak cross-tolerance in their study. The +8°C heat shock (21–29°C) was used because peak temperatures of this magnitude have been observed in rock pools inhabited by *B*. *medius* on hot summer days [8]. The more extreme +10°C (21–31°C) heat shock was used to gauge how fish would cope with a future climate change scenario, where peak daytime temperatures may become higher in rock pools. Two measures of hypoxia tolerance were also employed. Firstly, the time to loss of equilibrium (LOE) under severe hypoxia (O_2_ = 7% air saturation) was measured. The hypoxia exposure of 7% air saturation was used as it is below previously reported critical oxygen tension (11% air saturation at 15°C; 25% air saturation at 25°C) for *B*. *medius* [13]. Thus, 7% air saturation was expected to be approximately 50% of critical oxygen tension at the temperature (21°C) used in the in the current study and represent a severe hypoxia challenge. As time to LOE is a standard proxy measure of hypoxia survival in fish [14,15], this allowed us to make comparisons to the findings of Todgham et al., (2005) [12] who demonstrated cross-tolerance as lower morbidity under hypoxia. It was therefore predicted that time to LOE under severe hypoxia would increase in *B*. *medius* after heat shock. Secondly, this study also measured the critical oxygen saturation (S_*crit*_) of *B*. *medius* after heat shock because, although it is a commonly used indicator of hypoxia tolerance in fish [16,17], S_*crit*_ was not measured by Todgham et al. (2005) [12]. S_*crit*_ represents the change in O_2_ consumption as fish transition from being an O_2_ regulator to an O_2_ conformer under progressive hypoxia exposure [16,17]. Among sculpins, species with a lower S_*crit*_ showed LOE after longer periods under exposure to severe hypoxia [14]. Therefore, under the expectation that *B*. *medius* would show evidence of cross-tolerance (longer time to LOE), it was predicted that S_*crit*_ would decline after heat shock. This would indicate that heat shock treated fish could maintain resting O_2_ consumption to lower O_2_ levels, and also provide insight as to whether cross-tolerance to hypoxia operates via mechanisms associated with modifications to the cardio-respiratory cascade. Despite the widespread use of S_*crit*_ as a measure of hypoxia tolerance in fish [16], to our knowledge, the effect of prior acute heat shock on S_*crit*_ following a period of recovery (~19 h) at ambient temperature has never been addressed.

## Materials and methods

All experiments were carried out at the Leigh Marine Laboratory, and the fish used were collected from rock pools (Hatfields Beach, Auckland, New Zealand, 36°34’S, 174°41’E) using hand nets. No access permit was required for the collection site as it is open to the public. Following capture, fish were housed in 30 L flow-through seawater tanks (air saturated, 200 μm filtered, 35 ppt salinity) and acclimated to constant temperature (~21°C), and a 12 h light-dark photoperiod for at least four weeks prior to experiments. Fish were fed daily on a mixture of crushed aquaculture feed (Skretting, Cambridge, TAS, Australia) and pilchard, and food was withheld for a period of 48 h prior to the start of experiments. All statistical analysis was performed using the Sigma Plot 13.0 software package and significance was accepted at P<0.05. The experiments were carried out under the approval of the University of Auckland Animal Ethics Committee (AEC approval number 001801) and all experiments were performed in accordance with relevant guidelines and regulations. Experiment 1 used LOE (i.e., the point when a fish can no longer maintain an upright position) as a proxy measure of survival. The fish used in this study maintained ventilation at LOE, and recovered rapidly (i.e. returned to an upright position) once returned to fully oxygenated conditions. As such, there was no mortality associated with LOE, and all animals were subsequently released to the wild. Nevertheless, fish were monitored daily for humane endpoints for 5 days following experimentation. The criteria for humane endpoints following a manipulation included: continued loss of equilibrium up to 24 h; loss of appetite and visible weight loss; prolonged erratic swimming, disorientation and tank collisions; and continued signs of infection, sores, or bleeding for up to 24 h. The planned method of euthanasia for any animal reaching humane endpoints and deemed irrecoverable was an overdose of anaesthetic (Aqui S 200mg/L). All authors have completed compulsory animal welfare training modules provided by the University of Auckland Animal Ethics Committee.

### Experiment 1: Cross-tolerance for survival of severe hypoxia following heat shock

Experiment 1 aimed to determine if a prior heat shock exposure influences the ability of the intertidal triplefin *B*. *medius* to survive severe hypoxia. The time to loss of equilibrium (LOE) under a constant severe hypoxia exposure (~7% of air saturation) was therefore measured in three experimental groups that either did or did not receive an initial heat shock. This included: (1) an ambient temperature (21°C) control group not exposed to heat shock (body mass = 2.13 g ± 0.14, N = 12), (2) a group exposed to a +8°C (~21–29°C) heat shock (body mass = 1.95 g ± 0.16, N = 12) and (3) a group exposed to a +10°C (~21–31°C) heat shock (body mass = 1.92 g ± 0.15, N = 12). There was no difference in body mass of fish in each experimental group (ANOVA, DF = 2, F = 0.56, P = 0.58). The heat shock exposure was carried out in a 50 L aquarium in which seawater was heated with aquarium heaters. At ~09:00, fish were transferred from holding tanks to the thermal ramping tank where they were held at 21°C for an hour. The water temperature was then gradually heated from 21°C, to either 29 or 31°C, over a 5 h period (see S1 Table for the exact rate of temperature change in each experimental run). The control group was handled identically to the +8°C and +10°C treatments, but instead of facing acute heating in the heat shock exposure aquarium, they were held at ambient temperature (21°C) for 5 h. After heat shock, fish were transferred to a 50 L tank supplied with ambient temperature (~21°C) flow through seawater. The fish were then allowed to recover overnight for ~19 h before being subjected to a hypoxic challenge (~7% of air saturation, see S1 Table). Hypoxia was achieved by bubbling seawater with N_2_ until ~7% of air saturation was reached (7–10 min of continuous bubbling). N_2_ was then bubbled in short bursts to maintain O_2_ at 7%. Fish were immediately removed from hypoxia once LOE occurred and placed in air saturated seawater to recover. Two groups of 6 fish were run separately for each of the three experimental treatments (i.e. a total of 6 experimental runs; see S1 Table for the details of each run).

### Experiment 2: Cross-tolerance for hypoxia following heat shock (critical oxygen saturation-S_*crit*_)

The purpose of experiment 2 was to determine if a prior heat shock exposure induced changes in the critical oxygen saturation (S_*crit*_) point of the intertidal triplefin *B*. *medius*. S_*crit*_ was assessed in three experimental groups: (1) an ambient temperature (21°C) control group not exposed to heat shock (body mass = 1.78 g ± 0.08, N = 10), (2) a group exposed to a +8°C (~21–29°C) heat shock challenge (body mass = 2.24 g ± 0.24, N = 10), and (3) a group exposed to a +10°C (~21–31°C) heat shock challenge (body mass = 2.06 ± 0.22, N = 8). There were no differences in body mass of fish in each experimental group (ANOVA, DF = 2, F = 1.65, P = 0.21), and the heat shock exposures were implemented following the same protocol as experiment 1. After heat shock, fish were transferred to respirometry chambers to assess S_*crit*_ using automated intermittent-flow respirometry (see McArley et al., (2018) [8] for detailed respirometry methods). Briefly, respirometers consisted of a cylindrical acrylic chamber fitted with an adjustable stopper, which allowed the chamber volume (60–110 ml) to be adjusted to match fish size. The inlet of each chamber was connected to an automated Eheim compact 3000 submersible flush pump (Eheim GmbH & Co. KG, Deizisau, Germany) controlled by custom-coded software (Leigh Marine Laboratory). An inline pump (modified Eheim compact 3000) was connected to the respirometry chamber in a closed loop to ensure adequate water mixing, and the oxygen concentration of water within the chamber was continuously measured using contactless sensor spots and FireSting O_2_ meters (PyroScience, Aachen, Germany). The R^2^ for the decline in O_2_ within the respirometer during the closed phase was >0.99 for the majority of measurement cycles and never <0.95.

The fish were first allowed to recover in the respirometers overnight at ~21°C (i.e., for 19 h after heat shock). During this recovery phase, mass specific oxygen consumption ($\dot{M}O_{2}$) was measured over repeated 8 min measurement cycles interspersed with 1 min flushing and wait periods (i.e. intermittent stop-flow respirometry). The standard metabolic rate (SMR) was estimated as the mean of the lowest 10% of $\dot{M}O_{2}$ values recorded in the overnight recovery period [18–20]. Following overnight recovery, a run of progressive hypoxia was initiated (~75, 55, 40, 30, 25, 20, 15, 10, 6% of air saturation) to resolve S_*crit*_ by identifying the oxygen saturation at which fish could no longer maintain $\dot{M}O_{2}$ above SMR [17]. Progressive hypoxia exposure began between 18–19.5 h following the conclusion of heat shock and was completed in ~4.5 h (see S2 Table). Temperature was maintained at ~21°C and N_2_ was bubbled into the water reservoir supplying respirometers to achieve each level of hypoxia. Three $\dot{M}O_{2}$ measurements were made at 75–20% of air saturation and one measurement was made at 15, 10 and 6% of air saturation. To determine S_*crit*_, SMR and $\dot{M}O_{2}$ at each level of hypoxia were plotted against water oxygen air saturation, and a linear regression forced through zero was performed between $\dot{M}O_{2}$ values which fell below SMR. The point where the regression line intersected with SMR was taken as the S_*crit*_ for an individual fish [21–24]. S_*crit*_ in each of the treatment groups was assessed over 3–4 separate experimental runs (see S2 Table for the details of each experimental run).

As background oxygen consumption was evident within the respirometry chambers by the end of experimental runs, background respiration for each measurement cycle was back calculated using linear regression and subtracted from $\dot{M}O_{2}$. The mean level of background respiration was equivalent to 4.7, 5.0, and 6.3% of $\dot{M}O_{2}$ in the control, +8°C heat shock and +10°C heat shock treatment groups respectively. There was no difference in the amount of background respiration among treatment groups (ANOVA, DF = 2, F = 2.18, P = 0.13).

### Statistics

In experiment 1, there were no differences in the time to LOE between experimental runs of the same treatment group, and data were pooled for subsequent analysis (N = 12 per treatment). Median loss of equilibrium time (LOE_50_) among treatments was assessed using Kaplan-Meier log-rank survival analysis and one-way ANOVA with Holm-Sidak *post-hoc* comparisons [15]. In experiment 2, there was a significant correlation between SMR and S_*crit*_ in all treatment groups (P = 0.004). Thus, analysis of covariance (ANCOVA) with Holm-Sidak post-hoc comparisons was used to determine if there were differences in S_*crit*_ among treatments. SMR was set as the covariate, and Holm-Sidak comparisons were used for *post-hoc* tests. SMR was compared among the control and heat shock treatments using one-way analysis of variance (ANOVA) with Holm-Sidak *post-hoc* comparisons. Statistical analysis was carried out using the SigmaPlot 14 and IBM SPSS Statistics 26 software packages, and significance was accepted at P<0.05.

## Results

### Experiment 1: Loss of equilibrium under a hypoxic challenge following acute heat shock

**Fig 1:**
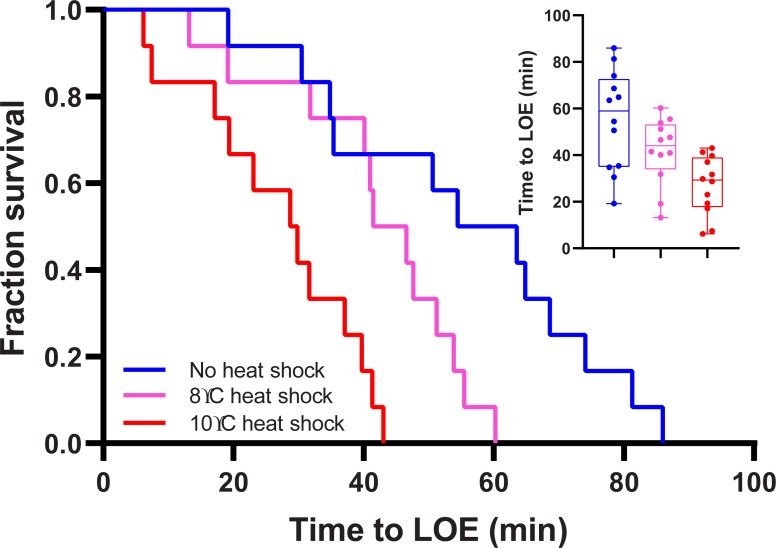
Survivorship curves and time to loss of equilibrium (LOE) in *Bellapiscis medius* exposed to severe hypoxia (7% air saturation) after 19 h recovery from acute heat shock. Blue line = control group (no heat shock), pink line = +8°C heat shock (21–29°C), red line = +10°C heat shock (21–31°C). Inset shows median time to loss of equilibrium and individual data points (N = 12 per treatment group). Different lower case letters in the inset indicate significant differences (P<0.05) in the median time to LOE among treatment groups.

During severe hypoxia exposure (~7% of air saturation) LOE occurred after 19–85 min in the control group, after 13–60 min in the +8°C heat shock group, and after 6–43 min in the +10°C heat shock group (Fig 1). The survival curves for time to LOE were significantly different among treatment groups (Log-Rank test, DF = 2, Statistic = 19.52, P<0.001). *Post-hoc* comparisons showed the median time to loss of equilibrium was significantly shorter in the +8°C heat shock group (LOE_50_ = 41.5 min) compared to the control group ((LOE_50_ = 54.4 min), and significantly shorter again in the +10°C heat shock group (LOE_50_ = 28.7 min) compared to both the control and +8°C heat shock group (Fig 1).

### Experiment 2: Critical oxygen saturation (S_*crit*_) following acute heat shock

**Fig 2:**
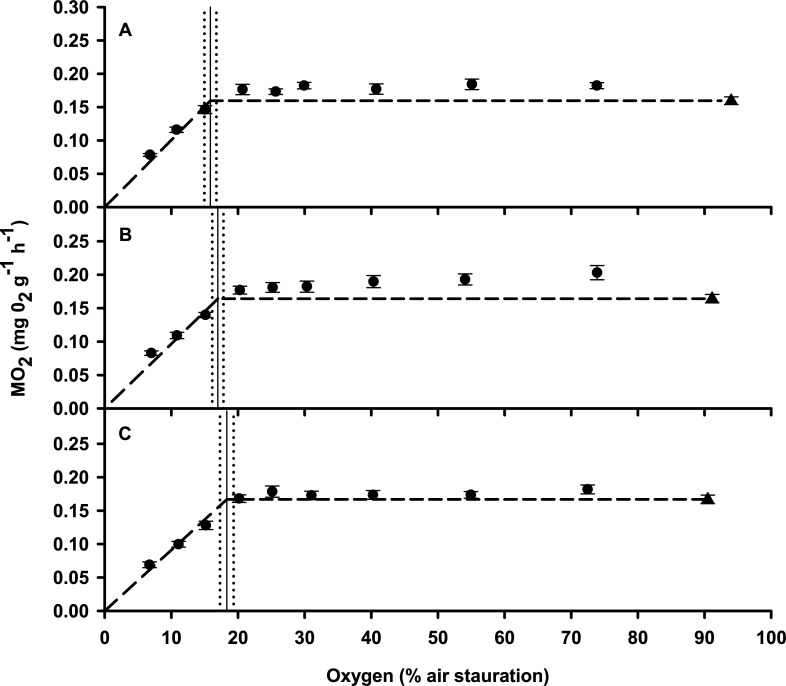
Critical oxygen saturation (S_*crit*_) and standard metabolic rate (SMR) of *Bellapiscis medius* following recovery from acute heat shock. All values represent mean ± S.E.M. A = control (N = 10), B = +8°C heat shock (N = 10), C = +10°C heat shock (N = 8). Triangles show SMR under normoxia, circles show routine $\dot{M}O_{2}$ under progressive hypoxia exposure, vertical line shows S_*crit*_ and vertical dotted line shows S_*crit*_ S.E.M. The dashed horizontal line shows the break point between oxygen regulation and oxygen conformation and is for illustrative purposes only.

The $\dot{M}O_{2}$ of fish was elevated in all treatment groups following entry to respirometers but had fallen to within 1 standard deviation of SMR after 8–11 h recovery. After overnight recovery in the respirometers, there was no difference in SMR between the control fish (0.16 mg O_2_ g^-1^ h^-1^ ± 0.005) and the fish exposed to an +8°C (0.16 mg O_2_ g^-1^ h^-1^ ± 0.006) or +10°C heat shock (0.16 mg O_2_ g^-1^ h^-1^ ± 0.005) (ANOVA, DF = 2, F = 0.184, P = 0.83, Fig 2). $\dot{M}O_{2}$ remained stable in all treatment groups during progressive hypoxia before declining abruptly below SMR between 15 and 20% of air saturation (Fig 2). The S_*crit*_ of the control fish was 15.8% of air saturation (± 0.9), but was slightly higher in fish exposed to an 8°C (S_*crit*_ = 17% of air saturation ±0.85) or 10°C (S_*crit*_ = 18.3% of air saturation ± 1.03) heat shock (Fig 2). There was, however, no significant difference in S_*crit*_ between any of the treatments (ANCOVA, DF = 2, F = 2.36, P = 0.12).

## Discussion

Knowledge relating to the impacts of multiple stressors is crucial to understanding the tolerance of marine organisms to real life environmental conditions [1–3]. The majority of studies have applied stressors simultaneously to assess organismal tolerance to multiple stressors, but this does not always represent ecologically relevant conditions because in many habitats different stressor types occur asynchronously [1]. Todgham et al., (2005) [12] sequentially exposed rock pool sculpins to high temperature then hypoxia, and provided evidence of cross-tolerance as fish showed improved hypoxia tolerance 8 h after recovering from the initial heat shock. Sequential stressor exposures are highly relevant for intertidal fish because, in rock pools, critically high temperatures can occur with intense solar radiation during daytime low tides, but then during the next low tide (~8–11 h later) severe hypoxia can develop under cooler night-time conditions [7–9]. However when examining the response of *B*. *medius*, a rock pool specialist within the family of New Zealand triplefin fishes, to sequential high temperature then hypoxia exposure we found no evidence of heat shock induced cross-tolerance.

Based on evidence of cross-tolerance from the study of Todgham et al., (2005) [12], our predictions were that the LOE_50_ of *B*. *medius* under severe hypoxia would increase, and that the S_*crit*_ would decrease after recovery from acute heat shock (i.e. *B*. *medius* would be preconditioned). However, neither prediction was supported because the LOE_50_ of *B*. *medius* decreased and S_*crit*_ showed no significant change. These findings therefore reveal no evidence for cross-tolerance in *B*. *medius* after acute exposure to peak temperatures observed in rock pools. In fact, the direction of change in LOE_50_ suggested that the hypoxia tolerance of *B*. *medius* was impaired by an initial heat shock. We cannot, however, discount the possibility that cross-tolerance is induced following exposure to less extreme heat shock (i.e. less than +8°C). Indeed, Todgham et al. (2005) [12] found the tolerance of sculpins to high salinity improved after a +12°C heat shock but not when a +15°C shock was applied. Thus, there may be a heat shock intensity threshold which, if surpassed, prevents the development of cross-tolerance. In the current study, the magnitude of heat shock exposures was based on field observations of temperature extremes in rock pools occupied by *B*. *medius* [8]. Moreover, fish were subjected to a thermal ramping protocol (i.e. increased temperature over 5 h) in order to replicate environmentally realistic heat shock in rock pools, where temperature gradually increases to a peak throughout the duration of low tide emersion. Thus, the concern raised by the current study is that *B*. *medius* showed a reduced tolerance to hypoxia after heat shock representative of current day scenarios (+8°C heat shock treatment) and hypoxia tolerance was further impaired in near-future warming scenario (+10°C heat shock treatment). This is a cause-for-concern as climate change is predicted to increase the frequency and intensity of heat wave events [25], which may result in more extreme rock pool temperatures in the near future and render *B*. *medius* less capable of coping with multiple stressors across successive low tides. On top of more extreme heat wave events, climate change involves chronic increases in mean environmental temperature occurring over prolonged time periods (i.e., decades). Physiological plasticity and/or adaptive capacity in the face of chronic warming may allow species to acclimate or adapt and gain additional resilience to climate change [26]. Thus, to better understand the vulnerability of *B*. *medius* to global warming, future studies should assess the influence of chronic warming on tolerance to hypoxia and acute high temperatures.

The S_*crit*_ of fish subjected to acute heat shock remained unchanged, clearly demonstrating there is no adjustment in the capacity of *B*. *medius* to meet basal O_2_ demands under low O_2_ following overnight recovery from heat shock. S_*crit*_ is determined by an interaction between the extractive capacity of a fish’s cardio-respiratory system for O_2_ and minimal resting metabolic demand for O_2_ (i.e. SMR) [27,28]. Since S_*crit*_ and SMR were similar across treatments, prior exposure to heat shock neither enhanced, nor hindered the extractive capacity of the cardio-respiratory system for O_2_. Yet, despite no differences in S_*crit*_, the LOE_50_ of *B*. *medius* under exposure to severe hypoxia (~7% air saturation) was still reduced after heat shock. It is important to note, however, that severe hypoxia in the current study exposed fish to an O_2_ level approximately 50% of S_*crit*_. The ability of an organism to survive hypoxia below S_*crit*_ partly relies on utilising anaerobic ATP production to maintain energy balance [28]. Thus, a reduced ability to survive levels of hypoxia below S_*crit*_ might reflect either faster energy expenditure and/or lower availability of endogenous fermentable fuel stores such as glycogen. Since there was no difference in SMR between treatment groups a faster rate of energy expenditure in heat shock exposed fish seems unlikely. As blood lactate is raised in fish exposed to acute high temperatures [29], it is possible heat shock treated fish lost equilibrium faster because tissue glycogen stores were diminished when they faced hypoxia ~19 h later. Further studies assessing tissue glycogen dynamics following acute heat shock, however, are required to substantiate this hypothesis.

The studies of Hilton et al., (2008; 2010) [13,30] demonstrate a 1.7–2.1 fold increase in the S_*crit*_ of *B*. *medius* with an acute increase in temperature from 15°C to 25°C over 2 h. However, in both these previous studies, hypoxia tolerance was assessed when the 25°C (i.e. +10°C) heat shock was applied, so the large increases in S_*crit*_ were likely due to elevated metabolic costs at high temperatures. As shown by McArley et al., (2018) [8], an acute +10°C increase in rock pool temperature is only likely to occur during daytime low tides associated with direct solar radiation and high air temperatures, conditions which also promote algal mediated hyperoxia due to photosynthesis. Thus, while the results of Hilton and colleagues demonstrate that *B*. *medius* suffers a substantial impairment of S_*crit*_ when hypoxia and acute heat shock co-occur, this is probably a circumstance rarely, if ever, faced under natural conditions.

Whilst cross-tolerance was not seen in the current study it is important to discuss two limitations in our experimental design that may have impacted our findings. *B*. *medius* in this study were allowed ~19 h to recover from heat shock before facing hypoxia but, in reality, fish undergoing a natural tidal cycle in the wild would experience a shorter time in which to recover from heat shock. For example, in one rock pool inhabited by *B*. *medius*, nocturnal O_2_ levels fell to 20% air saturation approximately 10 h after the peak in daytime temperature [8]. We therefore cannot discount the possibility that a shorter 10 h recovery period after acute heat shock might reveal cross-tolerance in *B*. *medius* where it was absent in the current study. In reality, it is more likely that a shorter recovery would lead to greater level of cross-sensitivity, so future studies should replicate the natural temporal pattern of multiple stressor exposures with greater precision if a complete, ecologically relevant result is to be formed. A second limitation is that rock pools occupied by *B*. *medius* also become hyperoxic during acute high temperature events [8]. This is important because, when facing an acute heat challenge combined with hyperoxia, the aerobic metabolic scope of *B*. *medius* is not constrained to the same degree as it is under normoxia [8]. It is thus possible that an expanded aerobic scope under hyperoxia during heat shock could dampen the requirement for anaerobic metabolism, thus preserving tissue glycogen stores and permitting fish to survive longer during a subsequent hypoxic exposure. We note that hyperoxia mitigated the rise in plasma and muscle lactate levels in two Antarctic notothenioid fishes exposed to acute heat shock [31], but clearly future research is required to resolve this issue fully.

The present study shows that prior exposure to heat shock impairs the subsequent hypoxia tolerance of the intertidal triplefin *B*. *medius*, thus providing no evidence of heat shock induced cross-tolerance to hypoxia in this species. Instead, *B*. *medius* appears to be mildly cross-sensitive to hypoxia after acute heat shock exposure (+8°C) representative of a current day situation. Perhaps more concerning, a +10°C heat shock representing a future climate change scenario results in a more significant sensitisation to hypoxia. Overall, *B*. *medius* is likely to retain relatively normal levels of hypoxia tolerance under current day conditions, but if heat shock events intensify with climate change as expected, this might substantially reduce the ability of this species to tolerate multiple ecologically relevant stressors.

## Supporting information

S1 File Supporting data (LOE, S_*crit*_, and SMR). (XLSX)

S1 Table Experiment 1 supporting information. (DOCX)

S2 Table Experiment 2 supporting information. (DOCX)
