# Supplementary material for: Acute high temperature exposure impairs hypoxia tolerance in an intertidal fish
Source: PLoS One. 2020 Apr 2;15(4):e0231091. doi: 10.1371/journal.pone.0231091 (PMC7117701; doi:10.1371/journal.pone.0231091)
Supplement: S1 Table — (DOCX) [file pone.0231091.s002.docx]

*Supporting information for article:* **Acute high temperature exposure impairs hypoxia tolerance in an intertidal fish**

Tristan. J. McArley^1*^, Anthony J.R. Hickey^2^ and Neill. A. Herbert^1^

^1^Institute of Marine Science, University of Auckland, Leigh, New Zealand

^2^ School of Biological Sciences, University of Auckland, Auckland, New Zealand

^*^Author for correspondence ([tmca008@aucklanduni.ac.nz](mailto:tmca008@aucklanduni.ac.nz))

S1 Table. Characteristics of individual experimental runs in experiment 1. TR= thermal ramping heat shock.

| Treatment group | Mean LOE time ± S.E.M. (min) | TR magnitude (°C) | TR time length (min) | TR heating rate (°C min^-1^) | Time to hypoxic exposure post TR (hh:mm) | Time to reach hypoxic set point (mm:ss) | O_2_ saturation during hypoxic challenge (% air saturation) | | | Temperature during overnight recovery (°C) | | | Temperature during hypoxic challenge (°C) | | |
| --- | --- | --- | --- | --- | --- | --- | --- | --- | --- | --- | --- | --- | --- | --- | --- |
| Ambient no HS (N=12)  Run 1 (*n=6*)  Run 2 (*n=6*)  +8°C HS (N=12)  Run 1 (*n=6*)  Run 2 (*n=6*)  +10°C HS (N=12)  Run 1 *(n=6*)  Run 2 (*n=6*) | 53.06 (10.16)  57.47 (7.98)  40.86 (5.5)  42.75 (6.67)  26.29 (5.24)  27.8 (5.54) | na  na  8.04 (21.01-29.05)  8.27 (20.79-29.06)  10 (21.05-31.05)  10.08 (21.01-31.09) | na  na  301  296  295  299 | na  na  0.027  0.028  0.034  0.034 | 19:16  19:18  19:15  19:20  19:15  19:21 | 09:27  10:19  09:37  07:04  07:36  09:06 | *Mean*  7.02  6.88  6.98  6.78  6.99  6.85 | *Min*  5.19  5.47  5.66  5.41  5.7  5.46 | *Max*  8.79  8.37  8.3  8.1  8.59  8.13 | *Mean*  21.03  20.72  20.88  21.18  21.04  21.05 | *Min*  20.42  20.21  20.18  20.34  20.31  20.44 | *Max*  21.68  21.52  21.59  21.82  21.81  21.75 | *Mean*  21.02  21.07  21.06  21.12  21.03  21.04 | *Min*  20.91  20.97  20.96  20.98  20.93  20.92 | *Max*  21.14  21.19  21.23  21.28  21.12  21.15 |
