# Supplementary material for: Acute high temperature exposure impairs hypoxia tolerance in an intertidal fish
Source: PLoS One. 2020 Apr 2;15(4):e0231091. doi: 10.1371/journal.pone.0231091 (PMC7117701; doi:10.1371/journal.pone.0231091)
Supplement: S2 Table — (DOCX) [file pone.0231091.s003.docx]

*Supporting information for article:* **Acute high temperature exposure impairs hypoxia tolerance in an intertidal fish**

Tristan. J. McArley^1*^, Anthony J.R. Hickey^2^ and Neill. A. Herbert^1^

^1^Institute of Marine Science, University of Auckland, Leigh, New Zealand

^2^ School of Biological Sciences, University of Auckland, Auckland, New Zealand

^*^Author for correspondence ([tmca008@aucklanduni.ac.nz](mailto:tmca008@aucklanduni.ac.nz))

S2 Table. Characteristics of individual experimental runs in experiment 2. TR= thermal ramping heat shock.

| Treatment group | TR magnitude (°C) | TR time length (min) | TR heating rate (°C min^-1^) | Post TR time period to progressive hypoxia exposure (h) | Total time length of progressive hypoxia exposure (min) | Temperature during SMR assessment (°C) | | | Temperature during P*_crit_* assessment (°C) | | |
| --- | --- | --- | --- | --- | --- | --- | --- | --- | --- | --- | --- |
| Ambient no HS (N=10)  Run 1 (*n=4*)  Run 2 (*n=4*)  Run 3 (*n=2*)  +8°C HS (N=10)  Run 1 (*n=1*)  Run 2 (*n=3*)  Run 3 (*n=4*)  Run 4 (*n=2*)  +10°C HS (N=8)  Run 1 *(n=3*)  Run 2 (*n=2*)  Run 3 (*n=1*)  Run 4 (*n=2*) | na  na  na  7.83 (21.2-29.03)  8.1 (20.97-29.07)  7.99 (20.97-28.96)  8 (21.02-29.02)  10.16 (20.84-31)  10.21 (20.87-31.08)  10.01 (21.1-31.11)  10.02 (21.03-31.05) | na  na  na  300  313  310  282  284  289  309  286 | na  na  na  0.027  0.026  0.026  0.029  0.036  0.036  0.033  0.036 | 18.33  18.76  18.56  18.85  18.1  18.33  19.5  19  19.1  18.5  19.4 | 292  271  267  274  261  270  267  273  266  272  291 | *Mean*  20.58  21.36  20.95  20.6  21.5  20.7  20.8  20.98  21.09  20.97  20.89 | *Min*  20.17  21.06  20.86  20.42  21.17  20.56  20.73  20.91  20.99  20.86  20.69 | *Max*  21.12  21.55  21.13  21.03  21.76  21.11  21.07  21.23  21.48  21.15  21.15 | *Mean*  21.12  21.19  21.12  20.99  21.09  21.24  21.07  21.2  21.22  20.99  20.99 | *Min*  20.92  20.99  21.01  20.89  21.05  20.96  20.95  21.12  21.11  20.89  20.94 | *Max*  21.3  21.37  21.33  21.04  21.22  21.35  21.27  21.24  21.29  21.07  21.04 |
